# Evolution of Cellular Organization Along the First Branches of the Tree of Life

**DOI:** 10.1007/s00239-024-10188-7

**Published:** 2024-07-17

**Authors:** Freya Kailing, Jules Lieberman, Joshua Wang, Joshua L. Turner, Aaron D. Goldman

**Affiliations:** 1 Department of Biology, Oberlin College, Oberlin, OH USA; 2 Blue Marble Space Institute of Science, Seattle, WA USA

**Keywords:** Last universal common ancestor, Last archaeal common ancestor, Last bacterial common ancestor, Cellular organization

## Abstract

Current evidence suggests that some form of cellular organization arose well before the time of the last universal common ancestor (LUCA). Standard phylogenetic analyses have shown that several protein families associated with membrane translocation, membrane transport, and membrane bioenergetics were very likely present in the proteome of the LUCA. Despite these cellular systems emerging prior to the LUCA, extant archaea, bacteria, and eukaryotes have significant differences in cellular infrastructure and the molecular functions that support it, leading some researchers to argue that true cellularity did not evolve until after the LUCA. Here, we use recently reconstructed minimal proteomes of the LUCA as well as the last archaeal common ancestor (LACA) and the last bacterial common ancestor (LBCA) to characterize the evolution of cellular systems along the first branches of the tree of life. We find that a broad set of functions associated with cellular organization were already present by the time of the LUCA. The functional repertoires of the LACA and LBCA related to cellular organization nearly doubled along each branch following the divergence of the LUCA. These evolutionary trends created the foundation for similarities and differences in cellular organization between the taxonomic domains that are still observed today.

**Supplementary Information:**

The online version contains supplementary material available at 10.1007/s00239-024-10188-7.

## Introduction

Cellular organization is a defining characteristic of all life on Earth. A detailed and accurate understanding of the emergence of cellularity is, therefore, crucial to our broader account of early evolutionary history. The subject, however, remains somewhat contentious due to countervailing lines of evidence, some of which indicates an early evolution of cellularity, perhaps even coincident with the origin of life, itself, and some of which indicates a late evolution of cellularity, perhaps even following the divergence of the LUCA into the separate ancestors of bacteria and archaea.

Protocell experiments have demonstrated that membranes can form spontaneously from prebiotically available compounds such as decanoic acid (Namani and Walde 2005) as well as the lipid fractions of carbonaceous chondrite meteorites (Deamer and Pashley 1989), suggesting a possible role for membrane compartmentalization as early as the origin of life, itself. Artificial life simulations have shown that protocell encapsulation could have protected early replicator genomes from parasites (Hogeweg and Takeuchi 2003; Takeuchi and Hogeweg 2009; Shah, et al. 2019) and supported genomic stability in general (Takagi, et al. 2020). Other artificial life simulations have also shown that, in the kind of rich environment often associated with the origin of life, selection would have acted against protocell encapsulation even if that encapsulation was imposed by the environment rather than produced by the life form, but that protocell encapsulation would have eventually co-evolved along with the first metabolic pathways (Szathmary 2007; Takagi, et al. 2020).

Phylogenetic reconstructions of universal paralog protein families have shown that cell membrane-associated proteins such as ABC transporters, ATP synthase enzymes, and the signal recognition particle system, all underwent gene duplications prior to the time of the last universal common ancestor (LUCA) (Gribaldo and Cammarano 1998; Kollman and Doolittle 2000; Zhaxybayeva, et al. 2005; Harris and Goldman 2021). This evidence would indicate that cellular organization was well in place by the time of the LUCA. However, the primary constituents of cell membranes, phospholipids, are radically different between archaea and bacteria (Pereto, et al. 2004; Sojo 2019). Typical bacterial phospholipids contain fatty acid chains that are connected to the phosphate head group by an ester linkage, while typical archaeal phospholipids contain isoprenoid chains that are connected to the head group by an ether linkage. Though all bacterial and most archaeal phospholipids are composed of a head group and two hydrophobic tails that form a bilayer membrane, some archaeal phospholipids contain hydrophobic tails that bridge across the bilayer with a phosphate head group on either side. These differences in the main constituents of cell membranes, as well as the metabolic pathways that produce them, have been taken as evidence by some researchers that true cellular organization evolved after the time of the LUCA (Wachtershauser 1988; Koga, et al. 1998; Martin and Russell 2003; Weiss, et al. 2016).

Given the different lines of evidence, some researchers have argued for a central role of protocell compartmentalization as early as the origin of life itself (for example, (Saha and Chen 2015; West, et al. 2017; Damer and Deamer 2020; Nunes Palmeira, et al. 2022; Goldman 2023)) and that the last universal common ancestor was a fully cellular organism (Becerra, et al. 2007; Goldman, et al. 2023), while others have argued that even by the relatively later stage of the LUCA, life forms were still not fully cellular (Wachtershauser 1988; Koga, et al. 1998; Martin and Russell 2003; Koonin and Martin 2005; Weiss, et al. 2016).

Here, we seek to reconcile the evidence for and against cellular organization by the time of the LUCA by identifying protein families associated with cellular organization in minimal proteome reconstructions of the LUCA as well as its successors, the last archaeal common ancestor (LACA) and the last bacterial common ancestor (LBCA). In doing so, we portray, in broad terms, the kinds of cellular functions that likely were and were not encoded in the LUCA and how these functions appear to have expanded along the first branches of the tree of life. By complementing an analysis of LUCA cellular functions with an analysis of the subsequent evolution of those functions following the LUCA, we aim to provide context for major differences in cellular organization between the bacteria and archaea that can still be observed today.

## Methods

Previously published minimal proteomes representing the LUCA (Crapitto, et al. 2022), the LACA (Williams, et al. 2017), and the LBCA (Coleman, et al. 2021) were modeled as EggNOG clusters of proteins (Huerta-Cepas, et al. 2019), a proxy for families of homologous proteins. These reconstructions are referred to as minimal proteomes because some protein families present in the actual proteome of each ancestor may have undergone subsequent loss or non-orthologous gene displacement and therefore would be absent from the ancestral proteome reconstruction (Koonin, et al. 1996). The LUCA minimal proteome (Crapitto, et al. 2022) was inferred through a meta-analysis of eight previously published LUCA proteome studies (Harris, et al. 2003; Mirkin, et al. 2003; Delaye, et al. 2005; Yang, et al. 2005; Ranea, et al. 2006; Wang, et al. 2007; Srinivasan and Morowitz 2009; Weiss, et al. 2016) and represents the consensus predictions between all eight studies. The LACA and LBCA minimal proteomes were inferred from tree reconciliation studies (Williams, et al. 2017; Coleman, et al. 2021) using the Amalgamated Likelihood Estimation (ALE) algorithm (Szollosi, et al. 2013), which compares gene and species trees to estimate gene duplications and losses as well as horizontal gene transfer.

Data from the LUCA, LACA, and LBCA minimal proteome studies were acquired from the supplementary information associated with each of the three studies. The minimal LUCA proteome consisted of 366 EggNOG clusters, the minimal LACA proteome consisted of 174 EggNOG clusters, and the minimal LBCA proteome consisted of 397 EggNOG clusters. Gene Ontology (GO) terms (Ashburner, et al. 2000; Gene Ontology, et al. 2023) associated with each EggNOG cluster were identified using the UniProt ID Mapping database (Bairoch and Apweiler 1997; Huang, et al. 2011; UniProt 2023).

For each ancestral proteome, EggNOG clusters associated with cellular function were identified by searching for the following GO terms related to cellularity: “membrane” (GO:0016020), “phospholipid biosynthetic process” (GO:0008654), “phospholipid transfer to membrane” (GO:0006649), “protein targeting” (GO:0006605), “endocytosis” (GO:0006897), “exocytosis” (GO:0006887), “transmembrane transporter activity” (GO:0022857), “cell division” (GO:0051301), “cytoskeleton” (GO:0005856), “structural constituent of cytoskeleton” (GO:0005200), “FtsZ-dependent cytokinesis” (GO:0043093), “single organism reproductive process” (GO:0044702), “membrane fission” (GO:0090148), and “mitotic cell cycle process” (GO:1903047). After identifying EggNOG clusters associated with broadly defined cellular functions, we gathered all other GO terms associated with these EggNOG clusters (Supplementary Data 1) and removed non-cellular functions by hand. For each individual GO term, we also included all other GO terms that were more general (i.e., the parent terms of each term, the parent terms of those parent terms, etc*.*).

The Gene Ontology database contains annotations that are nested within more general annotations, or what the Gene Ontology database refers to as “parent” terms. For example, the GO term "SRP-dependent co-translational protein targeting to membrane" (GO:0006614) is nested within the more general parent GO term, “co-translational protein targeting to membrane” (GO:0006613), which itself is nested within the more general parent GO term "protein targeting to membrane" (GO:0006605). Different proteins within the LUCA, LBCA, and LACA, minimal proteomes were annotated at different levels of specificity and, overall, bacterial proteins tended to be annotated with more specific GO terms, while archaeal proteins tended to be annotated with more general GO terms.

**Fig. 1:**
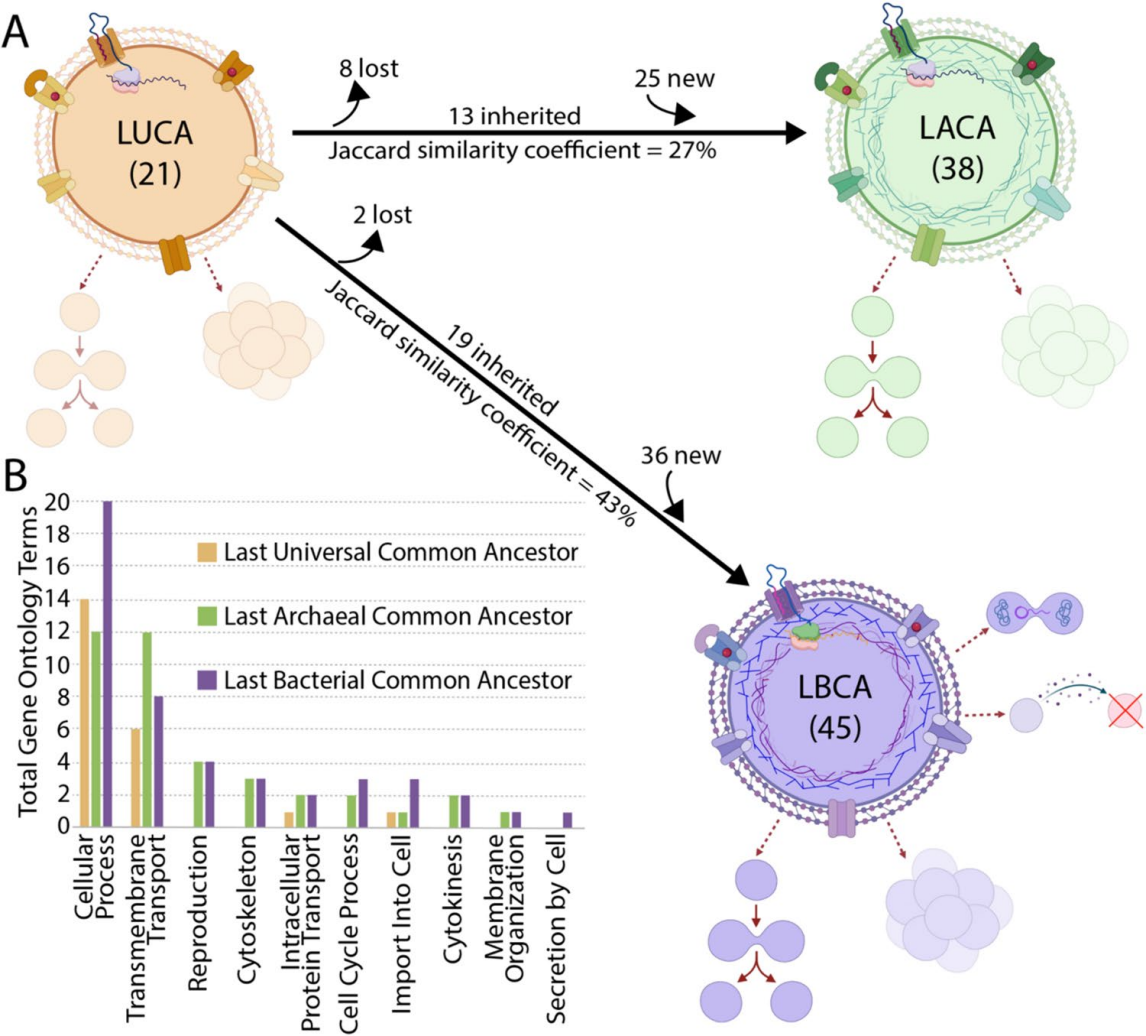
Cellular functions associated with the minimal proteomes of the LUCA, LACA, and LBCA. **A** The number of GO terms associated with cellular organization in each ancestor and the number of functions that are inferred to have been inherited, gained, and lost along each branch. Only GO terms that are direct child terms of the fifteen general GO terms relating to cellular organization were analyzed. **B** The total number of direct child GO terms within each category of general GO terms for each ancestor. Some of the original fifteen general GO terms produced redundant direct child GO terms and were combined. This figure was, in part, created with BioRender

In order to directly compare these datasets, we standardized the level of specificity across all three ancestral minimal proteomes so that the presence or absence of membrane-associated GO terms could be compared between the LUCA, LACA, and LBCA. To do so, we identified fifteen GO terms that appeared to be at a similar level of generality and then identified the direct child terms of these general GO terms. These general GO terms were “reproduction” (GO:0000003), “cytokinesis” (GO:0000910), “transporter activity” (GO:0005215), “cytoskeleton” (GO:0005856), “intracellular protein transport” (GO:0006886), “cytoskeleton organization” (GO:0007010), “cellular process” (GO:0009987), “asexual reproduction” (GO:0019954), “cell cycle process” (GO:0022402), “reproductive process” (GO:0022414), “reproduction of a single-celled organism” (GO:0032505), “secretion by cell” (GO:0032940), “transmembrane transport” (GO:0055085), “membrane organization” (GO:0061024), and “import into cell” (GO:0098657). These general GO terms and their direct child terms are used in the analysis described below. Several of these general GO terms produced redundant child terms and were combined, resulting in the ten general GO terms shown in Fig. 1.

## Results

Within the minimal proteomes of the LUCA, LACA, and LBCA, we found 39, 27, and 45, EggNOG clusters associated with membrane functions, respectively (Supplemental File 1). Most of these EggNOG clusters were identified using the “membrane” GO term: 33/39 LUCA EggNOG clusters, 18/27 LACA EggNOG clusters, and 33/45 LBCA EggNOG clusters, representing 11%, 16%, and 11%, of the protein families in each ancestor’s proteome, respectively. Very few EggNOG clusters were associated with the “phospholipid biosynthetic process” GO term: 4/39 LUCA EggNOG clusters, 1/27 LACA EggNOG clusters, and 1/45 LUCA EggNOG clusters. The primarily eukaryotic GO terms, endocytosis, exocytosis, membrane fission, and mitotic cell cycle process, did not yield any EggNOG clusters in the reconstructed proteomes of the LUCA, LACA, and LBCA.

We hierarchically standardized the collected GO terms in order to compare them across ancestral proteomes (see Methods). This hierarchical analysis resulted in fifteen parent terms and fifty-seven direct child terms (Supplemental File S2). Comparing the non-redundant sets of direct child terms between the LUCA and the LACA or LBCA shows that 90% of GO terms associated with the LUCA were also present in the LBCA dataset while 62% of GO terms associated with the LUCA were present in the LACA dataset. The Jaccard similarity index between the LUCA and the LBCA GO terms was 43%, while the Jaccard similarity index between the LUCA and the LACA GO terms was 27%. The Jaccard similarity index between two datasets is calculated as the intersection, i.e., the number of shared items, divided by the union, i.e., the total number of items in both datasets. As a measure of similarity, it therefore takes into account both the number of shared items between two datasets and the sizes of both datasets. By both methods of comparison, i.e., the percentage of shared GO terms in the LUCA dataset and the Jaccard similarity index, the LBCA appears closer to the LUCA in terms of cellular functions, while the LACA appears more distinct from the LUCA.

The cellular functions associated with each ancestor and the differences between them are shown in Fig. 1. Most cellular functions associated with the LUCA proteome involve transport across the membrane, including the transport of amino acids, carbohydrates, and ions. The LUCA proteome also encodes the ability to target proteins to the membrane, a function that would be required for embedding transporter proteins into the membrane in the first place. Other functions associated with the reconstructed LUCA proteome are related to cell division, cell motility, cellular response to stimuli, cell wall organization or biogenesis, and cell aggregation.

Comparisons between cellular functions associated with the reconstructed LUCA proteome and that of its successors, the LACA and LBCA, are largely characterized by the introduction of new cellular functions with a small number of functions lost along each branch (Fig. 1). These new functions acquired by the LACA and LBCA following the divergence of the LUCA suggest a parallel evolution of new cytoskeletal elements and cell reproduction processes as well as differentiation with respect to specific membrane transport functions. The LBCA ancestral proteome also includes functions associated with horizontal gene transfer (GO:0009292) as well as the killing of other cells (GO:0001906) through toxin activity (GO:0090729).

## Discussion

Inferring the proteomes of organisms that lived approximately 3.5-4Gya is inherently difficult (Crapitto, et al. 2022). EggNOG clusters may be incorrectly included or excluded from one of the ancestral proteomes due to limitations of the methodologies. For example, a protein family may have been present in the LUCA but lost to such an extent in subsequent lineages that it cannot be reconstructed as such. For simplicity, we describe differences in the presence and absence of protein functions between the three ancestral proteomes as gains and losses, but these results can also be explained by methodological shortcomings. For this reason, we caution against interpreting the results of an individual protein family being present in one of the ancestral proteomes as definitive. Instead, we portray the evolution of cellular functions in the LUCA, LACA, and LBCA in broad terms that both address the competing hypotheses about when cellularity first evolved and also provide a roadmap for future research.

Taken together, these results suggest that the LUCA represents a population of cellular organisms. By the time of the LUCA, many different cellular functions had evolved, and these appear to have expanded during the subsequent evolution of the LACA and the LBCA. The small number of phospholipid biosynthesis enzyme families found in all three datasets agrees with the observation that bacterial (and eukaryotic) phospholipids differ chemically from archaea. Recent evidence suggests that phospholipid chemistry is diverse even within the archaeal (Caforio and Driessen 2017) and bacterial (Sohlenkamp and Geiger 2016) domains, which explains the lack of conserved phospholipid biosynthesis enzymes even within the LACA and LBCA proteomes. However, despite the lack of a clear signal of conserved phospholipid biosynthesis in any of these ancestors, other cellular systems were clearly in place at the time of the LUCA (Lombard, et al. 2012) and expanded upon during the evolution of the LACA and LBCA.

The cellular functions associated with the minimal LUCA proteome depict a cellular organism capable of embedding proteins within the membrane and controlling, to some extent, the translocation of ions and biomolecules across that membrane. Importantly, the LUCA also seems to have been capable of controlling its cell division rather than relying on spontaneous growth and division as is observed in protocells (Berclaz, et al. 2001; Hanczyc, et al. 2003). The LUCA also appears to have had at least some form of a cell wall even though cell wall composition is not universal across the bacteria, archaea, and eukaryotes. The LBCA and LACA appear to have evolved additional functions related to transmembrane transport and cell reproduction and exhibit a parallel evolution of cytoskeletal elements. However, it is also possible that any or all of these features were present in the LUCA as well, but were not reconstructed as such by our methods. The LBCA also evolved several other traits including cell killing through toxins and the ability to actively facilitate horizontal gene transfer, suggesting that it lived within a complex microbial ecology (Goldman and Kacar 2023).

Perhaps most intriguingly, LUCA appears to have more cellular functions in common with the LBCA than the LACA, suggesting that cellular organization in the LUCA was more like that of bacteria than archaea. If this trend is also true for phospholipid biosynthesis, it would imply that the LUCA membrane was composed of bacteria-like phospholipids, i.e., fatty acid tails and an ester-linked phosphate head group, and that the archaeal phospholipids with isoprenoid tails and an ether-linked phosphate head group were derived in the LACA lineage following the divergence of the LUCA. Future studies pairing phylogenetic analysis with ancestral reconstruction will provide greater detail about specific protein families present in the LUCA, LACA, and LBCA, as well as the molecular functions that those proteins were performing in ancient life.

## Supplementary Information

Below is the link to the electronic supplementary material.

Supplementary file1 (XLSX 104 KB)

Supplementary file2 (XLSX 10 KB)
